# The effect of interactive digital interventions on physical activity in people with inflammatory arthritis: a systematic review

**DOI:** 10.1007/s00296-018-4010-8

**Published:** 2018-03-19

**Authors:** Alison J. Griffiths, Claire M. White, Peter K. Thain, Lindsay M. Bearne

**Affiliations:** Faculty of Life Sciences and Medicine, School of Population Health and Environmental Sciences, King’s College London, Addison House, Guys Campus, London, SE1 1UL UK

**Keywords:** Inflammatory arthritis, Rheumatoid arthritis, Juvenile idiopathic arthritis, Physical activity, Interactive digital intervention

## Abstract

The aim of this systematic review was to evaluate the evidence from randomised controlled trials (RCTs) evaluating the effectiveness of interactive digital interventions (IDIs) for physical activity (PA) and health related quality of life (HRQoL) in people with Inflammatory Arthritis [rheumatoid arthritis (RA), juvenile idiopathic arthritis (JIA) axial Spondyloarthritis (AS) and psoriatic arthritis (PsA)]. Seven electronic databases identified published and unpublished studies. Two reviewers conducted independent data extraction and quality assessment using the Cochrane risk of bias tool (RoB). The primary outcome was change in objective PA after the intervention; secondary outcomes included self-reported PA and HRQoL after the intervention and objective or self-reported PA at least 1 year later. Five manuscripts, reporting four RCTs (three high and one low RoB) representing 492 (459 RA, 33 JIA) participants were included. No trials studying PsA or AS met the inclusion criteria. Interventions ranged from 6 to 52 weeks and included 3–18 Behaviour Change Techniques. Due to heterogeneity of outcomes, a narrative synthesis was conducted. No trials reported any significant between group differences in objective PA at end of intervention. Only one low RoB trial found a significant between group difference in self-reported vigorous [MD Δ 0.9 days (95% CI 0.3, 1.5); *p* = 0.004], but not moderate, PA in people with RA but not JIA. There were no between group differences in any other secondary outcomes. There is very limited evidence for the effectiveness of IDIs on PA and HRQoL in RA and JIA and no evidence for their effectiveness in PsA or AS.

**Electronic supplementary material:**

The online version of this article (10.1007/s00296-018-4010-8) contains supplementary material, which is available to authorized users.

## Background

Physical activity (PA) and exercise are key life-long strategies for the management for people with Inflammatory Arthritis (IA) [Rheumatoid Arthritis (RA), Psoriatic Arthritis (PsA), Axial Spondyloarthritis (AS) and Juvenile Inflammatory Arthritis (JIA)] and are recommended in clinical guidelines [1–4] to manage symptoms, disability and co morbidity [5–7].

Current public health recommendations advise that adults should complete at least 150 min of moderate PA, or 75 min of vigorous PA, or equivalent per week as well as twice weekly strengthening exercise [8]. Children are recommended to undertake considerably more activity of at least 60 min of moderate to vigorous activity per day, with vigorous activity completed on at least 3 days per week [9]. People with IA should aim to achieve these recommendations but take into account baseline activity level, disease activity and symptoms and incorporate therapeutic exercise prescriptions [10–12].

However, adherence to PA in people with IA tends to be low [13, 14] and there are complex and distinctive barriers which hamper PA participation [15, 16]. Personal (e.g., past exercise behaviour) physical (e.g., pain, fatigue), social and psychological (e.g., motivation) [17–19] factors may all influence PA participation and introducing potentially burdensome lifestyle changes to increase PA, is challenging [20, 21].

Restricted resources and increasing demand means access to face-to-face healthcare interventions to support PA uptake and maintenance is limited [22], consequently, novel ways to increase PA participation are needed. Interactive digital interventions (IDIs) use information and communication technology to combine health education with support to promote behaviour change by enabling interaction with healthcare practitioners [23, 24]. Such interventions may provide effective and efficient methods of supporting PA and have already shown promising results in changing health behaviours, such as supporting weight loss in obese adults [25] and smoking cessation [26]. However, changing behaviour is complex and requires the implementation of evidence-based principles [27]. The Medical Research Council recommends identifying and applying theory to inform behaviour change intervention design [28] and there is some evidence that theory informed interventions are associated with effectiveness [29, 30].

A range of IDIs have been developed for supporting self-management, including PA, for people with IA [31–33] and this systematic review evaluated the evidence from randomised controlled trials (RCTs) investigating the effectiveness of IDIs in people with Inflammatory Arthritis (IA) [rheumatoid arthritis (RA), juvenile idiopathic arthritis (JIA) axial spondyloarthritis (AS) and psoriatic arthritis (PsA)] on PA and health-related quality of life (HRQoL) after the intervention and at least 12 months.

## Methods

### Data sources

A comprehensive electronic database search for published [Medline (1946–2016 via Ovid), EMBASE (1947–2016 via Ovid), PsychInfo (2002–2016 via Ovid), Cinahl (1937–2016 via EBSCOhost), Cochrane Central Register of Controlled Clinical Trials (CENTRAL) and (PEDro 1929–2016) and unpublished (Open grey, http://www.opengrey.eu)] studies was conducted from the earliest records until July 2017. The final search was completed on 28th July 2017. Reference lists of relevant systematic reviews [33–36] and included studies were hand searched for additional eligible studies. No language or date restrictions were applied. Authors were contacted for further information, if required.

Search terms included MeSH, keyword and wild-card terms located in the title or abstract for three broad concepts reflecting the disease (e.g., IA), interventions or variables (e.g., IDIs) and outcome (e.g., Objective PA or self-reported activity) (Full search strategy in supplementary appendix A).

### Study selection

#### Eligibility criteria

Studies were included in this systematic review if they were RCTs that reported at least one measure of objective or self-reported PA and which met the following eligibility criteria:

1. Participants diagnosed with RA, PsA, AS or JIA diagnosed according to established criteria [37–40]. Studies were also included where a non-inflammatory or mixed population of participants were studied if the populations were reported separately [41].
2. Any intervention using an interactive digital intervention (IDI) which aimed to promote PA was included. For this review, IDIs are defined as interventions accessed through any digital platform (e.g., computers, smartphones or handheld devices, web based programmes, wearable technology or applications (apps)) that provides a self-management component and includes an interactive element that requires individuals to input personal data and engage with healthcare practitioners to obtain tailored feedback. This could include activity logs, goal setting, discussion forums, task reminders, or activity monitoring.
3. The study comparison groups comprised either: interventions not involving IDIs, e.g., information only (including information or advice delivered via a digital platform but with no interactive component), usual care (e.g., face to face interventions), or waiting list comparisons.

### Types of outcome measures

#### Primary outcome measure

Objectively measured PA or exercise capacity: measured from baseline to the end of the intervention period using a monitoring device, e.g., pedometer step count, accelerometry or other wearable technology, with data collected over at least 3 days, was considered. Outcomes could be reported as energy expenditure [Metabolic equivalent of task (METS)], time spent on PA or PA guideline achievement. Measures of exercise capacity such as maximal aerobic capacity (*VO*^2^ max) were included.

#### Secondary outcome measures

1. Self-reported PA: measured from baseline to the end of the intervention using any validated measured questionnaires, such as the International Physical Activity Questionnaire (IPAQ) [42] or PA diaries [43].
2. Health related quality of life (HRQoL): measured from baseline to the end of intervention using any validated tool, such as the Short form 36 (SF-36) [44].
3. Objective or self-reported PA: measured at least 1 year after the end of the intervention.

All citations identified from the searches were compiled using Endnote bibliographic software (EndNote X7.5.3). After the removal of duplicate records, all retrieved titles and abstracts were independently screened for inclusion by two researchers (AG, PT). The full text of eligible studies were examined independently for inclusion by two reviewers (AG, PT) using a bespoke screening tool that was designed and piloted a priori. Reviewers were not masked to the name(s) of the study author(s), institution(s) or publication source. Any disagreements were resolved by consensus.

### Data items and extraction

Data extraction was conducted by two independent reviewers (AG, PT) using a data extraction tool developed a priori (available on request). Participant demographics, intervention and control characteristics, the length of the intervention and follow up periods, pre- and post-intervention and follow-up outcome data for primary and secondary outcomes were extracted. Behaviour change techniques (BCTs) included in the interventions were also coded using the BCT taxonomy—version 1 [45] by two reviewers trained to identify BCTs using the taxonomy (AG, LB).

Data for outcomes reported at time points which were not the focus of this review were not included. Any discrepancies in data extraction were resolved by consensus. When consensus could not be reached another co-author (CW) served as arbitrator.

### Risk of bias in individual studies

Risk of bias was assessed independently by two reviewers (AG, PT) using the Cochrane Risk of Bias Tool [46]. This tool assessed risk of bias across six domains: random sequence generation and allocation concealment (both sources of selection bias), blinding of participants and personnel (performance bias), blinding of outcome assessment (detection bias), incomplete outcome data (attrition bias) and selective reporting (reporting bias). Studies are classified as having either the presence or potential presence of a source of bias (Yes), no risk of bias (No) or unclear risk of bias.

Sequence allocation, as reported by the study authors, was accepted as adequate where a variety of methods to account for age and sex were employed, including blocking, stratification, balancing and cluster randomisation. The determination of selective outcome reporting was limited to the stated primary and secondary outcomes only. Any discrepancies were resolved by consensus.

### Summary measures and planned statistical analysis

Since blinding of study personnel and participants to complex interventions is difficult this domain was not considered when rating overall risk of bias for individual studies. Therefore, studies were rated as having a high or low risk of bias if there was > 1, 1 or no sources of bias in addition to potential performance bias respectively.

In cases where a study had more than one intervention or comparison group, results from similar groups were combined for reporting [47].

Mean differences (MD) and 95% confidence intervals (CI) for between group change scores were calculated when possible, using Review Manager 5 Software (Version 5.3). If calculations were not possible due to missing data, the authors’ original results were presented.

Due to the clinical heterogeneity of population and outcome measures used by the included studies it was not possible to conduct meta-analyses. Therefore, a narrative synthesis of the included RCTs was conducted.

We have ensured, where possible, that we report this review in accordance with the preferred reporting items for systematic reviews and meta-analyses (PRISMA) guidance [48].

## Results

### Study selection characteristics

**Fig. 1:**
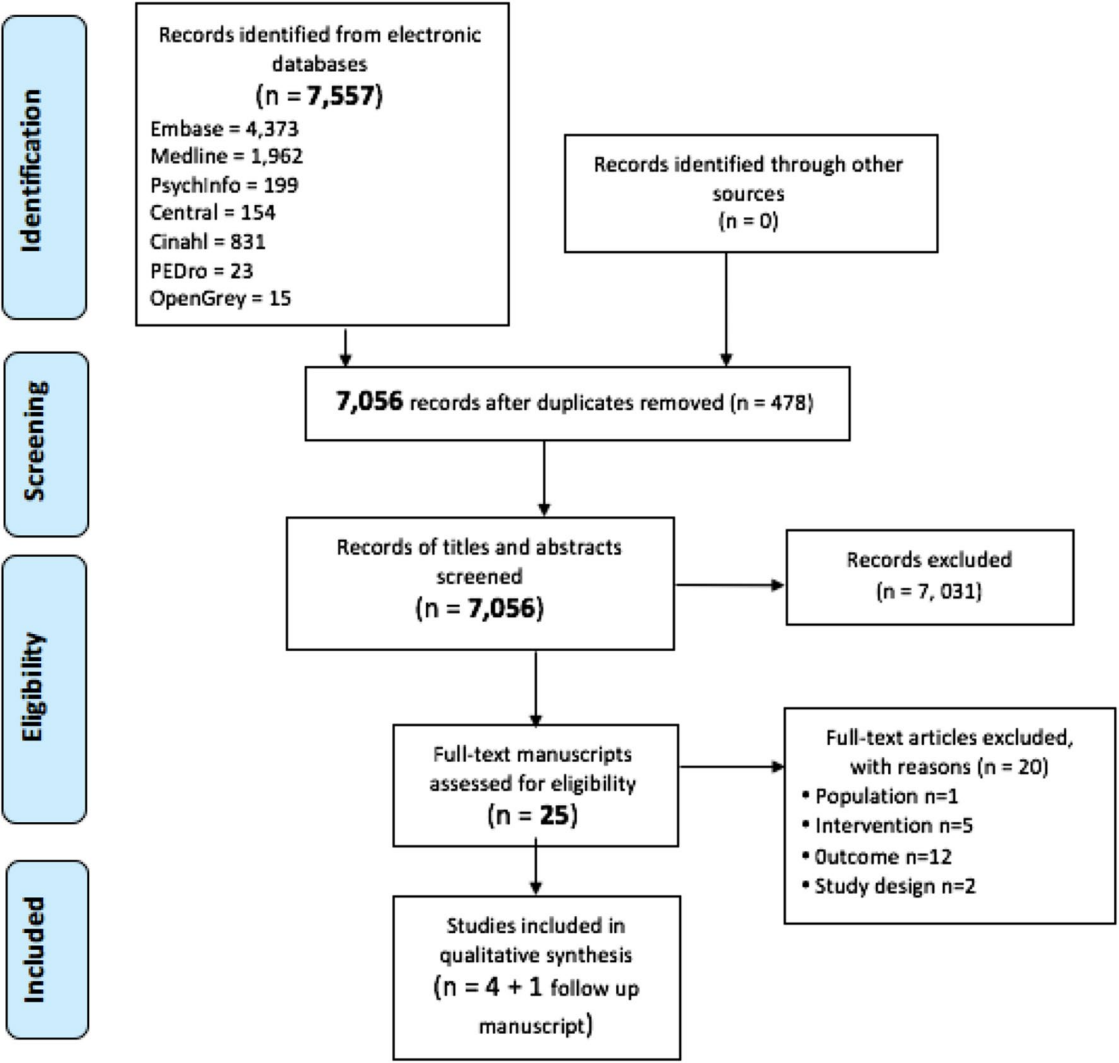
Flow diagram illustrating study selection

We identified 7557 potentially relevant citations. After removal of 478 duplicates, 7056 titles and abstracts were screened for eligibility. The full text of 25 studies were screened, of which five publications reporting four trials and one follow up study with a total of 492 participants were included in the review (Fig. 1). Studies were published between 2006 [33] and 2015 [49]. Two trials and one follow-up study were conducted in the Netherlands [33, 50, 51], one trial was conducted in Ticino (an Italian speaking part of Switzerland) [49] and one trial was completed in the USA [52]. No unpublished trials were included.

### Participants

There were a total of 492 included participants in four RCTs, with 110 of these participants re-assessed at 1 year after intervention cessation in a follow-up study [50]. One trial investigated people with JIA [51], two trials [33, 49, 50] and one follow up study [50] included only people with RA and one trial included people with RA, osteoarthritis and fibromyalgia, but the results from each population were reported separately at 1 year post-intervention cessation [52]. No included trials investigated PsA or AS.

Two trials [33, 51] and one follow-up study [50] reported no significant between group differences in participant sociodemographic characteristics. One trial, which included participants with different conditions, reported no overall significant between group differences in participant demographic characteristics, but did not report participant data with different conditions separately [52]. One trial did not report between group participant sociodemographic characteristics [49] (Table 1).

**Table 1:** Key characteristics of included studies

Study: First Author (date)	Participant characteristics Disease population (% female) number enrolled Age: Mean (SD) (years)	Intervention group characteristics# Theoretical Model Number (names) of behaviour change techniques	Comparison group characteristics	Outcome of interest Time points
Allam (2015) [49]	Adults with RA (45.8%) *n* = 155 (*I* = 85, *C* = 70) 57.95(12.29) years	Duration: 4 months ONESELF website: provides health, disease and PA Information and either (3 groups) 1. Social support – forum/chat room with 9 moderated sessions 2. Gaming—activities and rewards for logging on and participation 3. Social support + gaming BCTs *n* = 3 (social support (unspecified/ emotional), credible source)	1.Waiting list control group 2. ONESELF website: health, disease & PA Information only BCTs *n* = 1 (credible source)	Self-reported PA: minutes/week (Exercise behaviours scale) End of intervention
Lelieveld (2010) [51]	Children with JIA (87.8%) *n* = 33 (*I* = 17, *C* = 16) 10.7 (1.5) years	Duration:17 weeks. Rheumates @ work weekly progressive online PA programme with tailored feedback, plus 4 group sessions with family (Health Promotion Model [53]) BCTs *n* = 6 (goal setting, behavioural contract, feedback on outcomes of behaviour, information on antecedents/about others’ approval, pros and cons)	Waiting list control group BCTs *n* = 0	Objective PA: Aerobic exercise capacity (Bruce Treadmill Test) Self-reported PA: number of days/week with > 1 h mod-vig activity (activity diary)End of intervention
Lorig (2008) [52]	Adults with RA. (90%) *n* = 144 (*I* = 72, *C* = 72) 52.5 (12.2) years	Duration: 6 weeks ASMP online; weekly web based instruction, access to bulletin board and individual tools e.g., exercise logs, medication diaries and tailored exercise programmes (Social Cognition Model) [54] BCTs *n* = 5 (action planning, self-monitoring of behaviour, social support (unspecified), demonstration of the behaviour, reduce negative emotions)	Usual care control group BCTs *n* = 0	Self-reported PA: minutes/day (activity diary) 1 year after end of intervention
Van den Berg (2006) 33	Adults with RA (76%) *n* = 160 (*I* = 82, *C* = 78) 49.5 (12.9) years	Duration: 1 year Tailored online weekly exercise prescription: 5 days/week of progressive strengthening (3 × 10 reps/day), aerobic (cycling from 10 > 30 min day) and ROM (3 × 10 reps day) exercises & access to webpages, group forum &weekly individual supervision. BCTs *n* = 9 (monitoring of behaviour by others without feedback, self-monitoring of behaviour, feedback on outcomes of behaviour, social support (unspecified/ emotional), instructions on how to perform the behaviour, demonstration of the behaviour, graded tasks, adding objects to the environment)	Access to general online information and advice BCTs *n* = 2 (graded tasks, instructions on how to perform the behaviour)	Objective PA: overall PA score calculated using an accelerometer over 5 days Self-reported PA: Days/week mod. active for > 30 min and vigorous active > 20 min HRQoL: RAQoL End of Intervention
Hurkmans (2010) 50	Adults with RA (77%) *n* = 110 (*I* = 56, *C* = 45) 50.6 (13.1) years	As above	As above	Self- reported PA: days/week moderately active for > 30 min, Days per week vigorously active > 20 min 1 year after end of intervention

*RA* rheumatoid arthritis, *n* number, *I* intervention group, *C* comparison group, *BCTs* behaviour change techniques, *ASMP* arthritis self-management program, *HAQ* Health Assessment Questionnaire, *mod* moderate, *No*. number, *PA* physical activity, *RAQoL* rheumatoid arthritis quality of life, *reps* repetitions, *ROM* range of movement, *vig* vigorous

The mean age of participants ranged from 10.6 years [51] to 57.9 years [49]. Three trials reported participant mean disease duration between 5.5 years [33] and 14 years [49].

### Intervention characteristics

All interventions were interactive home-based website interventions [33, 49–52] and ranged from 6 [52] to 52 weeks [33] in duration. Interventions included provision of PA information [49] personalised exercise programmes [33, 50, 52] or tailored web-page summary of individual current PA, fitness and disease status reports [51]. Only one manuscript described the exercise programme recommended to participants in the intervention arm (Table 1) [33]. These were supplemented with discussion boards [33, 49, 52], regular e-mail communication between health practitioners and participants [33, 49–52] and /or face to face group or individual sessions [33, 50, 51] (Table 1).

Two trials [51, 52] investigated interventions explicitly underpinned by a theoretical model of behaviour change (Health Promotion Model [53]), Social Cognition Model [54] (Table 1). In total, 18 different BCTs were identified in the intervention arms of the included trials. Each intervention included at least three BCTs (range 3 BCTs [49])–9 BCTs [33] (Table 1). Unspecified Social Support was included in three interventions [33, 49, 52] and self-monitoring of behaviour [33, 52] and feedback on outcomes of behaviour [33, 51] were both included in two trials (Table 1).

### Comparison group characteristics

Comparison groups included waiting list control groups [51], usual care [52] or provision of information on exercise and physical activity guidelines [33, 49, 50]. No BCTs were included in two trials [51, 52], one trial used 1 BCT (credible source) [49] whilst one used 2 BCTs (graded task and instruction on how to perform the behaviour) [33].

### Primary outcome: objective measurement of physical activity at the end of the intervention

Objective PA was measured in two trials [33, 51]. One trial used an activity monitor for 3 days to calculate a general PA score which is expressed as the average number of accelerations in participant movement in a 5-min period [33]. Another trial assessed the change in aerobic exercise capacity as maximal endurance time during increasing walking speed and gradient using the Bruce treadmill test [51] (Table 1).

### Secondary outcomes self-reported measurement of physical activity at the end of the intervention

Three trials [33, 49, 51] measured participant self-reported PA. One trial used the exercise behaviour scale [43] to identify the mean number of minutes of PA per week [49], one trial used a PA diary to record the number of days that more than 1 h of moderate to vigorous PA was undertaken [51] and a further trial used a diary to identify the number of days per week that participants were either moderately active for more than 30 min or vigorously active for more than 20 min [33]. One trial measured diarised self-reported aerobic exercise (minutes/week), but did not report data from participants with different conditions separately at the end of the intervention [52] (Table 1).

### Physical activity at 1 year following cessation of the intervention

Two trials assessed self -reported PA at 1 year after the end of the intervention [50, 52]. Three trials did not collect any follow up data beyond immediately after cessation of the intervention [33, 49, 51]. No trials reported an objective measure of PA at least 1 year after the end of the intervention.

### Health related quality of life at the end of the intervention or 1 year following the end of the intervention

One trial reported HRQoL at the end of the intervention [33] using the rheumatoid arthritis quality of life (RaQoL) scale where a lower score indicates better quality of life [52].

### Risk of bias in included studies

**Fig. 2:**
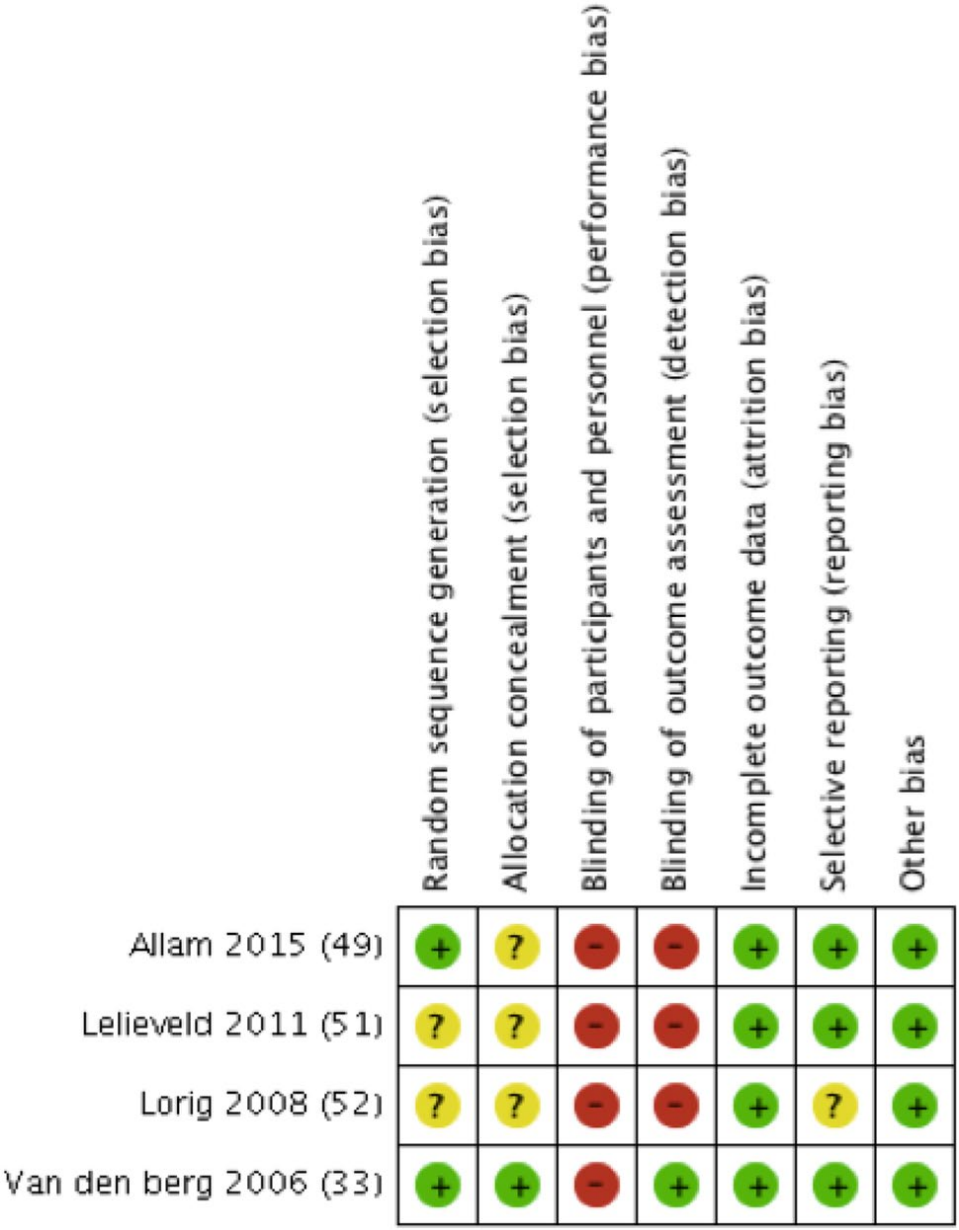
Risk of bias summary. Review authors’ judgements for each included study

Figure 2 summarises the sources of risk of bias for included studies. Two studies and one follow-up study reported adequate methods for random sequence generation [33, 49, 50], whereas two were unclear due to poor reporting [51, 52]. Reporting of allocation concealment was also unclear in three studies [49, 51, 52]. However, two of these reported no significant differences between groups for baseline characteristics [51, 52] although one was reported across a mixed population including participants with fibromyalgia and osteoarthritis in addition to those with IA [52]. Additionally, this study did not report results for each condition separately at the end of the intervention, but reported the findings for separate conditions at 1 year only [52].

Overall, there was evidence for the presence of high risk of bias in three studies [49, 51, 52], and low risk of bias in one trial [33] and one follow-up study [50] (Fig. 2).

### Primary outcome: objective physical activity at the end of the intervention

There were no significant between group differences in change in objective PA at the end of the intervention in one trial with low risk of bias including 155 participants with RA [33] and one trial [51] with high risk of bias including 33 participants with JIA (Table 2).

**Table 2:** Outcomes of interactive digital interventions on physical activity and health related quality of life at the end of the intervention and/or 12 months after the end of the intervention in people with inflammatory arthritis

Study	Outcomes			
	Physical Activity at the end of the intervention		Health related quality of life at end of intervention	Physical activity at 1 year after end of intervention
	Objective	Subjective		
Allam (2015) [49]	**–**	− 4.69 (− 13.36, 3.98) minutes/week^a^ *p* = 0.29	**–**	**–**
Lelieveld (2010) [51]	2s (− 48.79, 52.79) *p* = 0.94 maximal endurance time^a,d^	0.2 (− 0.92, 1.32) number of days/week of > 1 h of moderate to vigorous PA^a,d^ *p* = 0.73	**–**	**–**
Lorig (2008) [52]	**–**	**–**	**–**	− 8.92 (− 41.06, 23.22) *p* = 0.58 PA min/week
Van den Berg (2006) [33]	1.2 (− 0.77, 3.17) Physical activity^b^ *p* = 0.23	0.4 (− 0.41, 1.21) *p* = 0.33^b^ moderate PA 0.9 (0.3, 1.5) *p* = 0.003^b^* vigorous PA	− 0.7 (− 1.98, 0.58) *p* = 0.29 RAQoL^b^	**–**
Hurkmans (2010) [50]	**–**	**–**		Moderate PA:IT 19% vs GT 24% *p* = 0.48^c^ Vigorous PA:IT 7% vs GT 2% *p* = 0.2^c^

*p < 0.05

*s* seconds, *PA* physical activity, *RAQoL* rheumatoid arthritis quality of life

^a^Mean (95% confidence interval) in between group difference post scores

^b^Mean difference (95% confidence interval) in the between group change scores

^c^Post intervention odds ratio

^d^Results of 3 intervention arms combined and 2 comparison group arms combined

### Secondary outcomes – self-reported physical activity at the end of the intervention

One trial at low risk of bias [33], including 77 participants with RA, found a significant between group difference in change in vigorous PA of 0.9 days favouring the intervention group [MD 0.9 days (95% CI 0.3, 1.5); *p* = 0.003], but not for moderate activity [MD 0.4 (95% CI − 0.41, 1.21) *p* = 0.33].

There was no significant between group difference in the change in the number of minutes of PA/ week in one trial at high risk of bias, including 155 participants with RA [49] or in the between group difference in the number of days that children with JIA were moderately to vigorously active for more than 1 h per day in one trial with high risk of bias including 33 participants (Table 2).

### Objective or self-reported physical activity 1 year after the end of the intervention

No significant differences in between group changes in number of participants achieving the Dutch PA recommendations for moderate or vigorous activity were found in one trial with low risk of bias including 160 participants with RA 1 year after the end of the intervention [33]. Similarly, there was no significant between group difference in aerobic exercise capacity in one trial with high risk of bias including 144 participants with RA [52].

### Health related quality of life at the end of the intervention

There was no significant between group difference in change in HRQoL in the one trial at low risk of bias, including 77 participants with RA that evaluated it [33].

## Discussion

This systematic review of four RCTs and one follow-up study, including 459 adults with RA and 33 children with JIA, is the first to explore the effectiveness of IDIs for increasing PA in participants with common inflammatory conditions. Three trials were at high risk of bias [49, 51, 52] and only one trial at low risk of bias [33]. No trials reported any significant between group differences in objectively measured PA and only one trial of low risk of bias found a significant between group difference in self-reported vigorous but not moderate PA [33]. However, self-report measures may overestimate PA, particularly vigorous-intensity PA, when compared with objective measures of PA [55].

No trials reported significant between group differences in HRQoL. Surprisingly, the trials included in this review only enrolled people with RA or JIA as no trials including people with PsA or AS met our inclusion criteria and no studies included follow up beyond 12 months.

One explanation for the limited evidence for the effectiveness of IDIs on PA may be that only one trial specifically recruited participants with low PA [33]. As exercise and PA has a dose- response relationship, which is greatest in those who are inactive or low PA levels, targeting those with low PA levels may be important and result in greatest difference in our outcomes of interest [56].

Public Health England recognises the importance of digital innovation for promoting healthy lifestyle choices, such as PA [57]. Using IDIs could increase access to individually tailored, cost effective healthcare for underserved populations, including people with IA [58], and are easily individualised [59]. This review is important because it shows that there is a paucity of high-quality evidence evaluating the effect of IDIs on PA or HRQoL in adults with IA and children with JIA despite its acceptability and effectiveness for improving PA in the healthy population [59] so cannot be confidently recommended in the management of people with IA to increase PA.

The trials included in our review used online programmes [33, 49, 51, 52], supplemented with other forms of communication (e.g., emails, forums, or face to face group and /or individual meetings) [33, 49, 51, 52]. No included trials that met our eligibility criteria delivered IDIs via mobile applications, despite the popularity and availability of smartphones and wearable technology, which may not represent contemporary IDI usage. Published trial protocols are available, evaluating the effects of text messaging and mobile internet services on PA in IA [31, 60].

Interventions incorporating theoretically underpinned BCTs and multiple methods of communicating with participants are potentially most effective at facilitating changes in health related behaviour [30]. Only two included trials explicitly stated that they were underpinned by a theoretical model of behaviour change [51, 52]. However, all interventions included multiple BCTs (between 3 [49] and 9 [33] BCTs) even those without an explicit theoretical model of behaviour change. Interestingly, the trial with the lowest risk of bias incorporated the greatest number of BCTs and several methods of communication in the intervention [33] and this was the only trial which reported any benefit of IDIs on PA. Additionally, this trial found that those participants who had high levels of engagement with the intervention (75–100% website usage rate) had greater improvements [33]. This corresponds with findings from an earlier study where higher internet user engagement was significantly associated with improved self-management outcomes, including self-efficacy and reduced catastrophizing, in an arthritic population [61]. There is limited evidence to guide selection, number and dosage of BCTs to be included in interventions promoting adherence to health related behaviour change [62, 63]. Michie et al. suggests behaviour change interventions, particularly those with fewer techniques, can be effective in some populations [62]. Bishop et al. suggests that trials which reported the greatest intervention effects compared an active treatment group to control groups containing a low number of BCTs. All the trials in this review had low numbers of BCTs in the control group yet only one trial, which had low risk of bias, found a between group difference in self-report PA [63].

Surprisingly, only two trials included in this review measured HRQoL [33, 52] but found that there was no significant between group differences at the end of the intervention [33] or 1 year after the end of the intervention [52], reflecting studies in both the general [64] and self-reported arthritis populations [65]. This may be because HRQoL is a multifaceted concept; therefore, changing PA levels alone may not be sufficient to affect this outcome. Despite this, HRQoL remains a key patient outcome to evaluate management strategies and is considered of greater value to patients than clinical measures [66] and thus it may be an important focus when designing future IDIs.

This review has a number of strengths. The search strategy explored a range of databases for published and unpublished trials and no date or language restrictions were applied to minimise publication bias, which is a threat to validity [46, 67]. It included only RCTs which are considered the gold standard study design to evaluate intervention effects [68]. Rigorous risk of bias assessment that accounted for the impact of blinding at the level of individual outcomes was used. Intervention content was explored and described using a recognised behaviour change taxonomy which can aid the development of future interventions [45].

There are some limitations to this review. Only trials investigating IDIs in RA and JIA met our eligibility criteria, which limits the generalisability of our findings. Similarly, no trials investigating the use of mobile technologies or applications were included although ongoing trial protocols were identified. Only one trial fully described the PA dosage and progression recommended in the intervention, limiting conclusions [33].This review only investigated measures of PA and HRQoL, however, other outcomes such as disability, social support or participant satisfaction may be useful to explore the impact of IDIs on people with IA [69]. Other psychological factors such as self-efficacy and affective response following PA, may be important for the uptake and maintenance PA but these variables were not universally measured [69].

The findings of this review suggest that there is limited evidence from a small number of trials for the effect of IDIs on objective PA in people with RA or JIA after the intervention or at least 1 year. There is limited evidence from one low risk of bias trial on the effect of IDIs on PA in an RA population. The other trials in RA and JIA were at high risk of bias and no trials studied PsA or AS were included so our results cannot be generalised to the wider IA population or in the long term. High quality research is recommended before IDIs can be confidently included in the management of IA to increase PA. As adherence to PA tends to be low in people with IA [19, 70, 71] future research should aim to capture the effect of IDIs in the long term.

## Electronic supplementary material

Below is the link to the electronic supplementary material.

Supplementary material 1 (DOCX 25 KB)

Supplementary material 2 (DOCX 33 KB)
