# Supplementary material for: The effect of interactive digital interventions on physical activity in people with inflammatory arthritis: a systematic review
Source: Rheumatol Int. 2018 Mar 19;38(9):1623–34. doi: 10.1007/s00296-018-4010-8 (PMC6105152; doi:10.1007/s00296-018-4010-8)
Supplement: Supplementary file 2 — Supplementary material 2 (DOCX 33 KB) [file 296_2018_4010_MOESM2_ESM.docx]

S2: Individualized Search Terms

**Medline (ovid) Search Strategy**

| 1. exp arthritis, rheumatoid/ |
| --- |
| 2. ((rheumatoid or reumatoid or revmatoid or rheumatic or reumatic or revmatic or rheumat$ or reumat$ or revmarthrit$) adj3 (arthrit$ or artrit$ or diseas$ or condition$ or nodule$)).tw. |
| 3. exp Spondylitis, Ankylosing/ |
| 4. (ankylos$ or spondyl$).tw. |
| 5. Arthritis, Psoriatic/ |
| 6. (psoria$ adj2 (arthriti$ or arthropath$)).tw. |
| 7. Exp Arthritis, Juvenile/ |
| 8. (juvenile adj2 arthritis).tw. |
| 9. (inflamm* adj2 arthr*).tw |
| 10. or/ 1-9 |
| 11. exp Computer systems/ |
| 12. exp Computer-Assisted Instruction/ |
| 13. exp Telecommunications/ 14. exp Software/ |
| 15. exp Cell Phones/ |
| 16. Accelerometry/ |
| 17. ((computer-based or web-based) adj6 (therap* or education*)).tw |
| 18. (computer* or Internet* or telecommunication*).tw |
| 19. (interactive or online or on-line).tw |
| 20. (telemedicine or tele-medicine or telehealth or tele-health).tw |
| 21. (electronic health or ehealth or e-health).tw |
| 22. (mobile health or mhealth or m-health).tw |
| 23. ((health* or treat* or therap* or intervention* or assist* or selfmanag* or self-manag*) adj6 (computer* or technolog* or software or ICT)).tw |
| 24. (world wide web or www or web-based or email or e-mail).tw |
| 25. (telephone* or phone* or mobile* or cellphone* or app* or text* or SMS or smartphone*).tw |
| 26. (acceleromet* or pedomet*).tw |
| 27. or/11-26 |
| 28. Motor Activity/ |
| 29. Exp physical endurance/ |
| 30. Exp Physical exertion/ |
| 31. Exp exercise/ |
| 32. Exp Walking/ |
| 33. Exp Exercise Therapy/ |
| 34. Exp Health promotion/ |
| 35. Exp Physical fitness/ |
| 36. (habitual adj3 activit*).ti,ab |
| 37. Exercise.mp |
| 38. (physical* adj3 (fitness or activ*)).tw |
| 39. (selfmanag* or self-manag* or promot*).ti,ab |
| 40. or/28-39 |
| 41. 10 AND 27 AND 40 |

**EMBASE (ovid) Search Strategy**

| 1. exp rheumatoid arthritis/ |
| --- |
| 2. ((rheumatoid or reumatoid or revmatoid or rheumatic or reumatic or revmatic or rheumat$ or reumat$ or revmarthrit$) adj3 (arthrit$ or artrit$ or diseas$ or condition$ or nodule$)).tw. |
| 3. spondyloarthropathy/ |
| 4. (ankylos$ or spondyl$).tw. |
| 5. exp psoriatic arthritis/ |
| 6. (psoria$ adj (arthriti$ or arthropath$)).tw. |
| 7. Exp juvenile rheumatoid arthritis/ |
| 8. (juvenile adj2 arthritis).tw |
| 9. (inflamm* adj2 arthr*).tw |
| 10. or/ 1-9 |
| 11. exp computer system/ or computer/ |
| 12. exp Telehealth/ |
| 13. exp computer program/ |
| 14. exp telemedicine/ |
| 15. exp Internet/ |
| 16. exp mobile phone/ |
| 17. e-mail/ |
| 18. text messaging/ |
| 19. accelerometer/ or accelerometry/ |
| 20. mobile application/ |
| 21. ((computer-based or web-based) adj6 (therap* or treatment* or education*)).tw. |
| 22. (computer* or Internet* or telecommunication* or ICT).tw. |
| 23. (interactive or online or on-line).tw |
| 24. (telemedicine or tele-medicine or telehealth or tele-health).tw |
| 25. (electronic health or ehealth or e-health).tw. |
| 26. (mobile health or mhealth or m-health).tw. |
| 27. ((health* or treat* or therap* or intervention* or assist* or selfmanag* or self-manag*) adj6 (computer* or technolog* or software)).tw. |
| 28. (world wide web or www or web-based or email or e-mail).tw. |
| 29. (telephone* or phone* or mobile* or cellphone* or app* or text* or SMS or smartphone*).tw. |
| 30. (acceleromet* or pedomet*).tw |
| 31. or/11-30 |
| 32. exp physical activity/ |
| 33. exp Fitness/ |
| 34. exp endurance/ |
| 35. exp exercise/ |
| 36. exp walking/ |
| 37. exp kinesiotherapy/ |
| 38. exp health promotion/ |
| 39. health behavior/ |
| 40. physical fitness/ |
| 41. (habitual adj3 activit*).ti,ab |
| 42. Exercise.tw |
| 43. (physical* adj3 (fitness or activ*)).tw |
| 44. (selfmanag* or self-manag* or promot*).ti,ab |
| 45. (health adj2 behav*).tw |
| 46. or/32-45 |
| 47. 10 AND 31 AND 46 |

**PsychINFO (ovid) Search Strategy**

| 1. exp Rheumatoid Arthritis/ |
| --- |
| 2. ((rheumatoid or reumatoid or revmatoid or rheumatic or reumatic or revmatic or rheumat$ or reumat$ or revmarthrit$) adj3 (arthrit$ or artrit$ or diseas$ or condition$ or nodule$)).tw. |
| 3. (ankylos$ or spondyl$).tw. |
| 4. ((psoria$) adj2 (arthriti$ or arthropath$)).tw. |
| 5. (juvenile adj2 arthritis).tw |
| 6. 1 or 2 or 3 or 4 or 5 |
| 7. exp computers/ |
| 8. exp computer applications/ |
| 9. exp Computer software/ |
| 10. exp computer assisted therapy/ |
| 11. exp computer mediated communication/ |
| 12. exp digital computers/ |
| 13. exp human computer interaction/ |
| 14. exp Internet/ |
| 15. exp telecommunications media/ |
| 16. exp Telemedicine/ |
| 17. exp Cellular phones/ |
| 18. exp text messaging/ |
| 19. exp mobile devices/ |
| 20. ((computer-assist* or computer-based or web-based) adj6 (therap* or treatment* or education*)).tw,ot. |
| 21. (computer* or Internet* or telecommunication* or ICT).tw,ot. |
| 22. (interactive or online or on-line or telemedicin* or cellular phon* or mobil* phon*).tw,ot. |
| 23. (telemedicine or tele-medicine or telehealth or tele-health).mp |
| 24. (electronic health or ehealth or e-health).tw. |
| 25. (mobile health or mhealth or m-health).tw. |
| 26. ((health* or treat* or therap* or intervention* or assist* or selfmanag* or self-manag*) adj6 (computer* or technolog* or software)).tw. |
| 27. (world wide web or www or web-based or email or e-mail or online).tw. |
| 28. (telephone* or phone* or mobile* or cellphone* or app* or text* or SMS or smartphone*).tw. |
| 29. (acceleromet* or pedomet*).tw |
| 30. 7or 8 or 9 or 10 or 11 or 12 or 13 or 14 or 15 or 16 or 17 or 18 or 19 or 20 or 21 or 22 or 23 or 25 or 26 or 27 or 28 or 29 |
| 31. exp Activity Level/ |
| 32. exp Physical Activity/ |
| 33. exp Physical Fitness/ |
| 34. exp Exercise/ |
| 35. exp ‘Quality of Life’/ |
| 36. exp Physical Therapy/ |
| 37. exp Walking/ |
| 38. exp Health Promotion/ |
| 39. (habitual adj3 activit*).ti,ab |
| 40. (physical* adj3 (fitness or activ*).tw |
| 41. (selfmanag* or self-manag*).ti,ab |
| 42. ((physical adj2 therapy) or physiotherapy*).tw |
| 43. ((quality adj2 life) or QOL).tw. |
| 44. 31 or 32 or 33 or 34 or 35 or 36 or 37 or 38 or 39 or 40 or 41 or 42 or 43 |
| 46. 6 AND 30 AND 44 |

**CENTRAL Search Strategy**

| #1  MeSH descriptor Arthritis, Rheumatoid explode all trees |
| --- |
| #2 ((rheumatoid or reumatoid or revmatoid or rheumatic or reumatic or revmatic or rheumat* or reumat* or revmarthrit*) near/3 (arthrit* or artrit* or diseas* or condition* or nodule*)):ti,ab |
| #3 MeSH descriptor: [Spondylarthropathies] explode all trees |
| #4 arthritis or ankylosing next spondyl* or rheumatic next disease* or rheumatic next disorder* or rheumatic next condition*:ti,ab |
| #5 #1 or #2 or #3 or #4 |
| #6 MeSH descriptor: [Computer systems] explode all trees |
| #7 MeSH descriptor: [Computer-Assisted Instruction] explode all trees |
| #8 MeSH descriptor: [Telecommunication] explode all trees |
| #9 MeSH descriptor: [Software] explode all trees |
| #10 MeSH descriptor: [Cell phones] explode all trees |
| #11 MeSH descriptor: [Accelerometry] this term only |
| #12 ((computer-based or web-based) near/6 (therap* or education*)) |
| #13 (computer* or Internet* or telecommunication*) |
| #14 (interactive or online or on-line or telemedicin* or cellular phon* or mobil* phon*) |
| #15 (telemedicine or tele-medicine or telehealth or tele-health) |
| #16 (electronic health or ehealth or e-health) |
| #17 (mobile health or mhealth or m-health) |
| #18 ((health* or treat* or therap* or intervention* or assist* or selfmanag* or self-manag*) near/6 (computer* or technolog* or software)) |
| #19 (world wide web or www or web-based or email or e-mail or online) |
| #20 (telephone* or phone* or mobile* or cellphone* or app* or text* or SMS or smartphone*) |
| #21 (acceleromet* or pedomet*) |
| #22 #6 or #7 or #8 or #9 or #10 or #11 or #12 or #13 or #14 or #15 or #16 or #17 or #18 or #19 or #20 or #21 |
| #23 MeSH descriptor: [Motor Activity] this term only |
| #24 MeSH descriptor: [Physical Endurance] explode all trees |
| #25 MeSH descriptor: [Physical Exertion] explode all trees |
| #26 MeSH descriptor: [Exercise] explode all trees |
| #27 MeSH descriptor: [Walking] explode all trees |
| #28 MeSH descriptor: [Exercise Therapy] explode all trees |
| #29 MeSH descriptor: [Health Promotion] explode all trees |
| #30 (exercise or selfmanag* or self-manag*) .ti,ab |
| #31 (habitual near/3 activit*) .ti,ab |
| #32 (physical* near/3 (fitness or activ*)) .tw |
| #33 #23 or #24 or #24 or #26 or #27 or #28 or #29 or #30 or #31 or #32 |
| #34 #5 and #22 and #33 |

**CINAHL Search Strategy**

| S1 (MH "Arthritis, Rheumatoid+") OR (MH "Arthritis, Psoriatic") OR (MH "Spondylarthritis+") |
| --- |
| S2 (MH "Spondylitis, Ankylosing") |
| S3 (MH "Arthritis, Juvenile Rheumatoid") OR "juvenile arthritis or jia or juvenile idiopathic arthritis") |
| S4 (ankylos* or spondy*) |
| S5 ((rheumatoid or reumatoid or revmatoid or rheumatic or reumatic or revmatic or rheumat* or reumat* or revmarthrit*) W3 (arthrit* or artrit* or diseas* or condition* or nodule*)) |
| S6 S1 or S2 or S3 or S4 or S5 |
| S7 (MH “Computer systems+”) |
| S8 (MH “Computer-Assisted Instruction”) |
| S9 (MH “Computer communication networks+”) |
| S10 (MH “Telecommunications+”) |
| S11 (MH “Software+”) |
| S12 (MH “Cellular Phone+”) |
| S13 (MH “Electronic Mail+”) |
| S14 (MH “Text Messaging”) |
| S15 (MH “Accelerometry+”) |
| S16 ((computer-based or web-based) W6 (therap* or education*)) |
| S17 (computer* or Internet* or telecommunication*) |
| S18 (interactive or online or on-line or telemedicin* or cellular phon* or mobil* phon*) |
| S19 (telemedicine or tele-medicine or telehealth or tele-health) |
| S20 (electronic health or ehealth or e-health) |
| S21 (mobile health or mhealth or m-health) |
| S22 ((health* or treat* or therap* or intervention* or assist* or selfmanag* or self-manag*) W6 (computer* or technolog* or software or ICT)) |
| S23 (internet* or world wide web or www or web-based or email or e-mail) |
| S24 (telephone* or phone* or mobile* or cellphone* or app* or text* or SMS or smartphone*) |
| S25 (acceleromet* or pedomet*) |
| S26 S7 OR S8 OR S9 OR S10 OR S11 OR S12 OR S13 OR S14 OR S15 OR S16 OR S17 OR S18 OR S19 OR S20 OR S21 OR S22 OR S23 OR S24 OR S25 |
| S27 (MH “Motor Activity”) |
| S28 (MH “Physical Activity”) |
| S29 (MH "Physical Fitness+") |
| S30 (MH “Exercise”) |
| S31 (MH "Quality of Life+") |
| S32 (MH “Physical Therapy”) |
| S33 (MH "Walking+") |
| S34 (MH “Health Promotion+) |
| S35 AB (habitual adj3 activit*) |
| S36 AB exercise or physical activity |
| S37 AB (selfmanag* or self-manag*) |
| S38 S27 OR S28 OR S29 OR S30 OR S31 OR S32 OR S33 OR S34 OR S35 OR S36 OR S37 |
| S39 S6 AND S26 AND S38 |

**PEDro Search Strategy**

| **Abstract & Title**: Rheumatoid arthritis / internet / online / computer / software / smartphone / mobile / app / application / digital / SMS / text |
| --- |
| **Therapy**: n/a |
| **Problem**: n/a |
| **Body Part**: n/a |
| **Sub discipline**: n/a |
| **Topic**: n/a |
| **Method**: clinical trial |
| **Author/Association**: n/a |
| **Title only**: Rheumatoid arthritis / internet / online / computer / software / smartphone / mobile / app / application / digital / SMS / text |
| **Source**: n/a |
| **Published** since: n/a |

**Open Grey Search Strategy**

| (rheumatoid arthritis OR psoriatic arthritis OR ankylosing spondylitis OR JIA) AND (computer OR internet OR smartphone OR app OR mobile OR online OR software OR digital) AND (physical activity OR exercise OR fitness OR health) |
| --- |
